# Molecular Mechanisms Underlying Protective Role of Quercetin on Copper Sulfate-Induced Nephrotoxicity in Mice

**DOI:** 10.3389/fvets.2020.586033

**Published:** 2021-01-08

**Authors:** Xinyan Peng, Chongshan Dai, Min Zhang, Subhajit Das Gupta

**Affiliations:** ^1^College of Life Sciences, Yantai University, Yantai, China; ^2^College of Food Engineering, Ludong University, Yantai, China; ^3^Department of Internal Medicine, University of Texas Southwestern Medical Center, Dallas, TX, United States; ^4^College of Veterinary Medicine, China Agricultural University, Beijing, China

**Keywords:** copper, oxidative stress, inflammation, kidney, Nrf2 pathway, NF-κB pathway

## Abstract

Copper overload is an established cause of nephrotoxicity, but the precise molecular mechanism remains unknown. Our study aimed to investigate the molecular mechanism of copper sulfate (CuSO_4_)-induced nephrotoxicity and the protective effect of the natural compound quercetin using a mouse model. Mice were orally administered CuSO_4_ only (200 mg/kg per day), or co-administered CuSO_4_ (200 mg/kg per day) plus quercetin (25, 50, or 100 mg/kg per day), or quercetin only (100 mg/kg per day), or vehicle for 28 days. The blood and kidneys were collected for the examination of serum biomarkers, oxidative stress biomarkers, changes in histopathology and gene and protein expression. Our results show that quercetin supplementation attenuates CuSO_4_-induced renal dysfunction and tubular necrosis in a dose-dependent manner. Quercetin supplementation at 50 and 100 mg/kg significantly attenuated CuSO_4_-induced oxidative damage. Quercetin supplementation also inhibited the activities of caspases-9 and−3, and the expression of p53 and Bax mRNAs. Furthermore, quercetin supplementation markedly activated the expression of Nrf2 and HO-1 mRNAs, but inhibited the expression of NF-κB, IL-1β, IL-6, and TNF-α mRNAs. In conclusion, our results revealed that quercetin supplementation could inhibit CuSO_4_-induced nephrotoxicity in mice via the inhibition of mitochondrial apoptotic and NF-κB pathways and the activation of Nrf2/HO-1 pathway. Our study highlights quercetin as a potential candidate in treating copper overload-induced nephrotoxicity.

## Introduction

Copper (Cu) is a transition metal that functions as an essential trace element in the process of metabolism, growth, and development in human and animals (1). Cu plays a critical role in the activities of numbers of metalloenzymes, including dopamine beta-mono-oxygenase, cytochrome-c oxidase, and Cu, Zn-superoxide dismutase (1). In some countries, copper sulfate (CuSO_4_) has been widely used as a feed additive in animal production (2). However, copper overload could cause the tissue or organ damage in human and other mammals, affecting the liver, kidney, brain, lung, intestine, heart, and testis (3–10). Over the past decades, Cu overload was suggested to be associated with the development of Wilson's disease and other neurodegenerative diseases, which has raised concerns worldwide (7, 11).

The kidney is susceptible to copper toxicity due to its important role in the physiology of filtration. Cu overload results in compromised kidney function through renal phosphaturia, diminished glomerular filtration, proteinuria, and amino aciduria (12). It has been previously reported that oral administration of CuSO_4_ at the dose of 100 mg/kg per day for 30 days can cause marked renal damage in rats (4). Earlier studies also reported that the molecular mechanism of Cu nephrotoxicity involved oxidative stress, apoptosis and autophagy, which were characterized by various signaling pathways, including mammalian target of rapamycin (mTOR) pathway, nuclear factor kappa B (NF-κB) pathway, p53 pathway, and the endoplasmic reticulum (ER) stress pathway (13–15). In *in vitro* models, CuSO_4_-induced cell death is associated with increases in the levels of senescence, apoptosis, autophagy, mitochondrial damage, and excessive production of reactive oxygen species (ROS) (16). Recently, Alharbi and colleagues found that turmeric could protect CuSO_4_-induced nephrotoxicity in rats by inhibiting oxidative stress and apoptosis (14). However, to date, we still have limited therapeutic options to treat nephrotic tissue damage caused by Cu overload or poisoning. The development and discovery of novel drugs that treat or prevent Cu overload-induced nephrotoxicity remains an unmet need.

**Figure 1:**
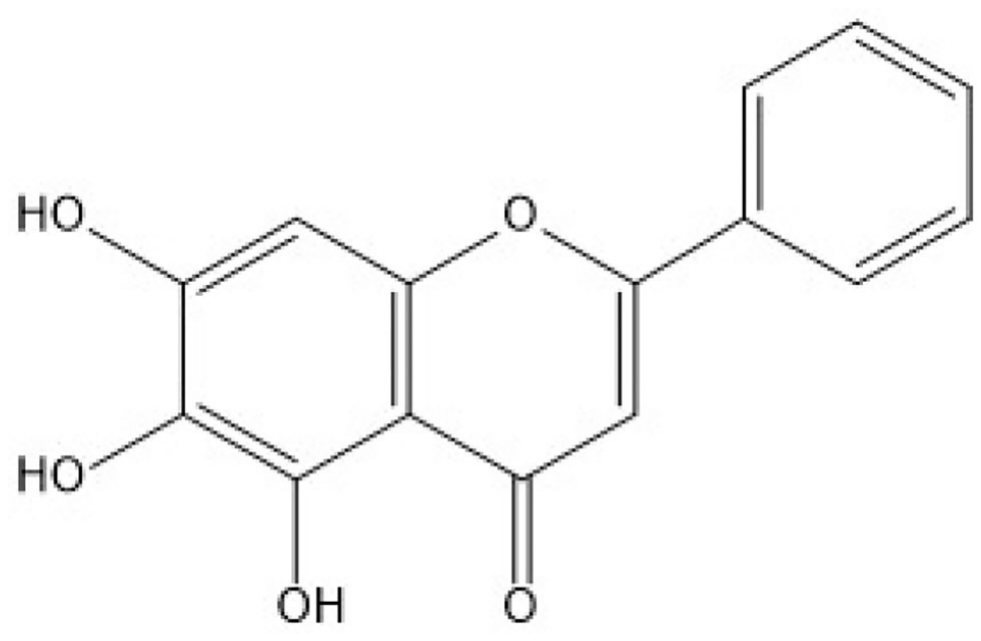
The chemical structure of the quercetin molecule.

Quercetin is a natural compound whose chemical formula is 3′, 3′, 4′, 5′, 7′-pentahydroxyflavone (Figure 1). Quercetin has many biological functions including anti-oxidant, anti-inflammatory, and immune-regulatory activities (17–19). Quercetin supplementation had been shown to attenuate D-Galactose-induced renal damage in rats and aflatoxin B1-induced neurotoxicity in mice through the inhibition of oxidative stress (20, 21). A recent study also showed that quercetin supplementation could inhibit lipopolysaccharide (LPS)-induced inflammation in a proximal tubular cell line human kidney 2 (HK-2) cells which derived from normal human kidney (22). Yet, whether quercetin may have a protective role in Cu overload-induced nephrotoxicity remains unknown. Therefore, in the present study, we investigated the potential protective effect of quercetin supplementation on CuSO_4_ exposure-induced nephrotoxicity in mice and the underlying molecular mechanisms.

## Materials and Methods

### Reagents and Chemicals

Quercetin (purity ≥98%) was bought from Aladdin (Shanghai, China). Copper sulfate (CuSO_4_•5H_2_O) was bought from Sinopharm (Shanghai, China). Carboxmethyl cellulose (CMC-Na) was obtained from Sigma-Aldrich (St. Louis, MO, USA). The standard diagnostic kits for blood urea nitrogen (BUN) and creatinine (CRE) were bought from Shanghai Kehua Bio-engineering Co., Ltd. (Shanghai, China). Phenylmethylsulfonyl fluoride (PMSF), aprotinin, pepstatin A, and leupeptin were purchased from AMRESCO Inc. (Ohio, USA). All chemicals were greater than or equal to analytical grade.

### Animals and treatments

All animal studies were approved by the Institutional Animal Care and Use Committee (IACUC) of Ludong University, Yantai, China (LDU-IRB20290503). Male C57BL/6 mice (8 weeks, 20–22 g) were purchased from Vital River Animal Technology, Co., Ltd. (Beijing, China). All mice had a 1 week acclimation period before experimentation. All mice were given the sufficient chow and water and housed under a light-dark period of 12 h, and a relative humidity of 50 ± 10%, and a temperature of 23 ± 2°C.

Sixty mice were split equally into the following 6 groups of 10 mice per group.

- control group;
- quercetin 100 mg/kg/day only (QUE100) group;
- CuSO_4_ 200 mg/kg/day only group;
- CuSO_4_ 200 mg/kg/day plus quercetin 25 mg/kg/day (QUE25 + CuSO_4_) group;
- CuSO_4_ 200 mg/kg/day plus quercetin 50 mg/kg/day (QUE50 + CuSO_4_) group
- CuSO_4_ 200 mg/kg/day plus quercetin 100 mg/kg/day (QUE100 + CuSO_4_) group

Control group mice were administered orally with equal volume of 0.5% CMC-Na (vehicle). Mice in all CuSO4 groups were administered orally with aqueous solution of CuSO4 at the dose of 200 mg/kg per day, in line with a previously published study (23). The previous studies reported that oral supplementation of quercetin in the range of 25–100 mg/kg/day for 1–4 weeks could protect against cadmium or gentamicin -induced renal toxicity in rats (24, 25). Therefore, the dose of quercetin at 25, 50, and 100 were used in the present study. Quercetin was dissolved in 0.5% CMC-Na and administered orally 2 h before CuSO_4_ administration. All sets of mice were treated with the respective chemicals for 4 weeks (e.g., 28 days). 24 h after the final dose, mice were humanely euthanized using sodium pentobarbital (80 mg/kg; intraperitoneal injection). Blood samples and kidney tissues were collected for histopathological, biochemical, and gene and protein expression examination, respectively.

### Measurements of Serum Markers Creatinine (CRE) and Blood Urea Nitrogen (BUN)

Blood samples were collected in 1.5 mL-tubes and centrifuged for 15 min at 3,000 × g, then the serum was isolated. The levels of BUN and CRE were determined by using Hitachi 7080 automated chemical analyzer (Hitachi High-Technologies Corporation, Tokyo, Japan).

### Histopathology Examination

Parts of kidneys were selected randomly from four mice. Tissues were fixed in 10% neutral-buffered formalin. After 48 h, kidney samples were used to prepare histological sections and hematoxylin-eosin (H&E) staining were performed. The histopathological scoring was done by a blind scorer using a semi-quantitative score (SQS) corresponding to the degree of renal tubular damage, following a previously published method (26).

### Ultrastructural Observation of Mitochondrial Morphometry in Kidney Tissues of Mice

Cortical sections of the murine kidneys were isolated and 1 mm cubes were cut for the ultrastructure analysis. In brief, samples fixed in 2.5% glutaraldehyde (buffered at pH – 7.2) were stored at 4°C overnight. After fixation, the samples were treated with 2% OsO_4_ dissolved in cacodylate buffer (0.1 M, pH 7.4, 4°C) for 2 h, then processed to dehydrate, embed, and slice. Finally, the ultrathin sections (70–80 nm) were stained with solutions of lead citrate and uranyl acetate. The images were taken by using a JSM25610LV transmission electron microscope (TEM) at a voltage of 100 kV.

### Measurements of Oxidative Stress Biomarkers

An amount of 50–100 mg of renal tissue was homogenized in 1 mL of homogenization buffer (0.1 mM Na-EDTA, 0.01 M Tris-HCl, 0.9% saline, 10 mM sucrose; pH 7.4) at 4°C. The supernatant was collected after centrifugation at 13,000 × g for 15 min at 4°C. The levels of glutathione (GSH), nitric oxide (NO) and malonaldehyde (MDA) and the activity of catalase (CAT), superoxide dismutase (SOD), and inducible nitric oxide synthase (iNOS) were determined by employing commercial MDA, GSH, NO SOD, CAT and iNOS assay kits (Nanjing Jiancheng, China). Total protein concentrations for all experiments were determined by using BCA assay kit (Beyotime,Haimen, China).

### Measurement of caspases-3,−9 activities

Caspases-3 and−9 activities in nephrotic tissue were examined by using the commercial caspase-3 and caspase-9 Assay kit (Beyotime, Haimen, China), respectively. Protocols were followed to the manufacturer's instructions.

### Measurement of TNF-α, Interleukin-1β (IL-1β), and Interleukin-6 (IL6) Levels in Renal Tissue

After treatment, the levels of IL-1β, TNF-α, and IL-6 in the renal tissues were measured by using IL-1β, TNF-α, and IL-6 enzyme-linked immunosorbent assay kits (R&D Systems, Minnesota, USA), as per manufacturer instructions.

### Western Blot

An amount of 10–20 mg of renal tissue was homogenized in 500 μL lysis buffer (0.1 M Tris-HCl, 2% SDS, 10% glycerol, 4°C; pH 7.4) with the mixture of protease inhibitor (1 mM PMSF plus the mixture of 1 μg/mL pepstatin A, 1 μg/mL leupeptin, and 1 μg/mL aprotinin). The supernatants were collected after centrifugation at 14,000 × g for 15 min at 4°C. Total protein levels were determined using a BCA assay kit. The primary antibodies used were against Bax (1:1,000), cleaved-caspase-3 (1:500), and GAPDH (1:5,000) (Cell Signaling Technologies, USA). The protein expressions were quantified using Image J software and normalized to the corresponding GAPDH bands.

### Quantitative Real-Time (qRT)-PCR

An amount of 30–50 mg of renal tissue was used to extract total RNA via the TRIZOL method (Life Technologies, Grand Island, NY, USA), as per manufacturer instructions. The integrity of collected RNA was checked by tabulating the ratio of the OD taken at 260 and 280 nm. Then, 1 microgram of total RNA from each tissue sample was used to reverse transcribe into cDNA using the Prime Script kit (Takara biotech, China). Quantitative real-time (qRT)-PCR reactions were carried out on the AB7500 instrument (Applied Biosystems, USA) and the expression of Nrf2, NF-κB, HO-1, IL-6, IL-1β, and TNF-α mRNA in the kidney tissue samples were measured and normalized to β-actin. The primer sequences used are listed as forward (top) and reverse (bottom) in Table 1.

**Table 1:** The primer sequences of quantitative real-time (qRT)-PCR.

**Gene name**	**Primer sequences (5^**′**^-3^**′**^) [forward (top) reverse(bottom)]**
p53	5**′**-AGAGTCTATAGGCCCACCCC-3**′**
	5**′**-GCTCGACGCTAGGATCTGAC-3**′**
Nrf2	5**′**-CACATTCCCAAACAAGATGC-3**′**
	5**′**-TCTTTTTCCAGCGAGGAGAT-3**′**
HO-1	5**′**-CGTGCTCGAATGAACACTCT-3**′**
	5**′**-GGAAGCTGAGAGTGAGGACC-3**′**;
NF-κB	5**′**-CACTGTCTGCCTCTCTCGTCT-3**′**
	5**′**-AAGGATGTCTCCACACCACTG-3**′**;
TNF	5**′**-AGCCGATGGGTTGTACCTTG-3**′**
	5**′**-ATAGCAAATCGGCTGACGGT-3**′**
IL1β	5**′**-CCGTGGACCTTCCAGGATGA-3**′**
	5**′**-GGGAACGTCACACACCAGCA-3**′**
IL-6	5**′**-AGGATACCACTCCCAACAGACCT-3**′**
	5**′**-CAAGTGCATCATCGTTGTTCATAC-3**′**
Bax	5**′**-AAACTGGTGCTCAAGGCCCT-3**′**
	5**′**-AGCAGCCGCTCACGGAG-3**′**
β-actin	5**′**-GCCCTGAGGCTCTTTTCCA-3**′**
	5**′**-GTTGGCATAGAGGTCTTTACGGAT-3**′**

### Statistics

All data are analyzed and represented as mean ± S.E.M. unless specified otherwise. Statistical analyses were performed on the SPSS V18.0 platform (SPSS Inc., IL, USA). A one-way ANOVA with Tukey's multiple comparison test was used when variance was homogenous; otherwise, Dunnett's T3 test was employed. A *P*-value lesser than 0.05 was set as statistical significance.

## Results

### Quercetin Supplementation Attenuated CuSO_4_-induced Kidney Dysfunction and Tubular Necrosis

**Figure 2:**
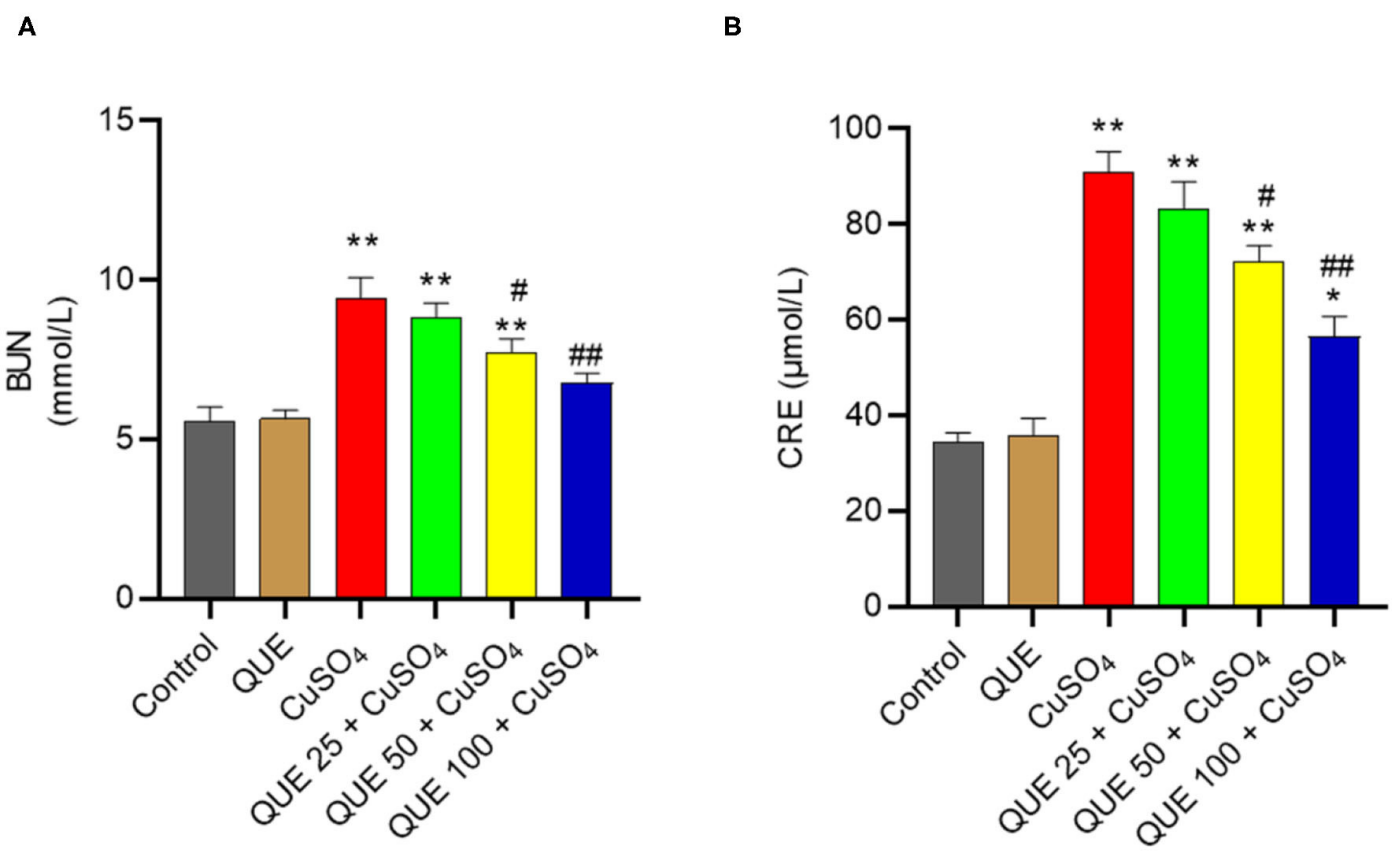
Effects of quercetin supplementation on the serum blood urea nitrogen (BUN) **(A)** and creatinine (CRE) **(B)**. Data are reported as mean ± S.E.M (*n* = 10). *P < 0.05, or **P < 0.01, compared to the untreated control group, respectively; ^#^*P* < 0.05, or ^##^*P* < 0.01 compared to CUSO_4_ alone group, respectively. QUE, quercetin.

Compared to the control group, serum BUN and CRE levels increased to 9.5 mmol/L and 91.2 μmol/L in the CuSO_4_ only group (both *P* < 0.01), respectively (Figure 2). Quercetin supplementation at the doses of 50 and 100 mg/kg/day significantly attenuated the CuSO_4_ exposure-induced increase in BUN and CRE levels (all *P* < 0.05 or 0.01). In the QUE50 + CuSO_4_ and QUE100 + CuSO_4_ groups, the serum BUN level decreased to 7.7 and 6.8 mmol/L, respectively (Figure 2A); the serum CRE level decreased to 72.1 and 56.6 μmol/L, respectively (Figure 2B).

**Figure 3:**
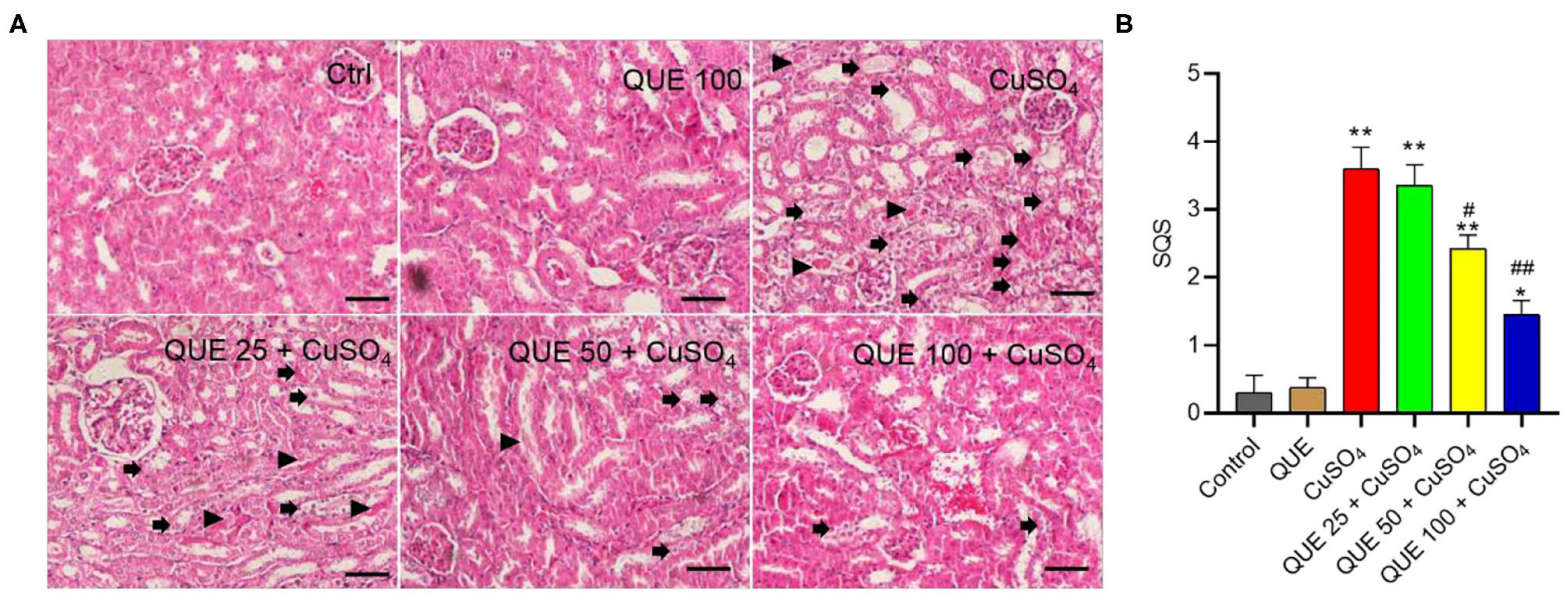
Representative histological sections of mouse kidney and the semi-quantitative scores (SQSs). **(A)** Observed histopathological changes of kidneys from control, QUE100 alone, CuSO_4_ alone, CuSO_4_ + QUE25, CuSO_4_ + QUE50, and CuSO_4_ + QUE100 groups. Black arrows indicate necrosis, tubular dilation and tubular degeneration; arrowheads indicate urinary cast formation. **(B)** SQSs are presented as the average ± S.E.M (*n* = 4). *P < 0.05, or **P < 0.01, compared to the untreated control group, respectively; ^#^*P* < 0.05, or ^##^*P* < 0.01, compared to the CuSO_4_ alone group, respectively. Hematoxylin-eosin staining. Bar = 50 μm. Ctrl, control; QUE, quercetin.

Quercetin supplementation also attenuated CuSO_4_-induced histopathological damages (Figure 3). Compared to the control group, marked tubular degeneration, tubular dilation, and necrosis were detected in the CuSO_4_ only groups, but these pathological changes were significantly lesser in the QUE50 + CuSO_4_ and QUE100 + CuSO_4_ groups (*P* < 0.05 or 0.01) (Figure 3A). In our scoring model, the SQS values decreased considerably from 3.6 to 2.4 and 1.5 in the QUE50 + CuSO_4_ and QUE100 + CuSO_4_ groups, respectively, compared to the CuSO_4_ alone group (Figure 3B). Quercetin supplementation at 25 mg/kg/day had no discernable effect on the biochemical parameters (i.e., BUN and CRE) and histopathological damages caused by CuSO_4_ exposure. There was no significant change in the levels of serum BUN and CRE or histopathological changes in the QUE100 alone group, compared to control group (Figures 2, 3).

### Quercetin Supplementation Attenuated CuSO_4_-induced Oxidative Stress in the Kidney Tissues

**Figure 4:**
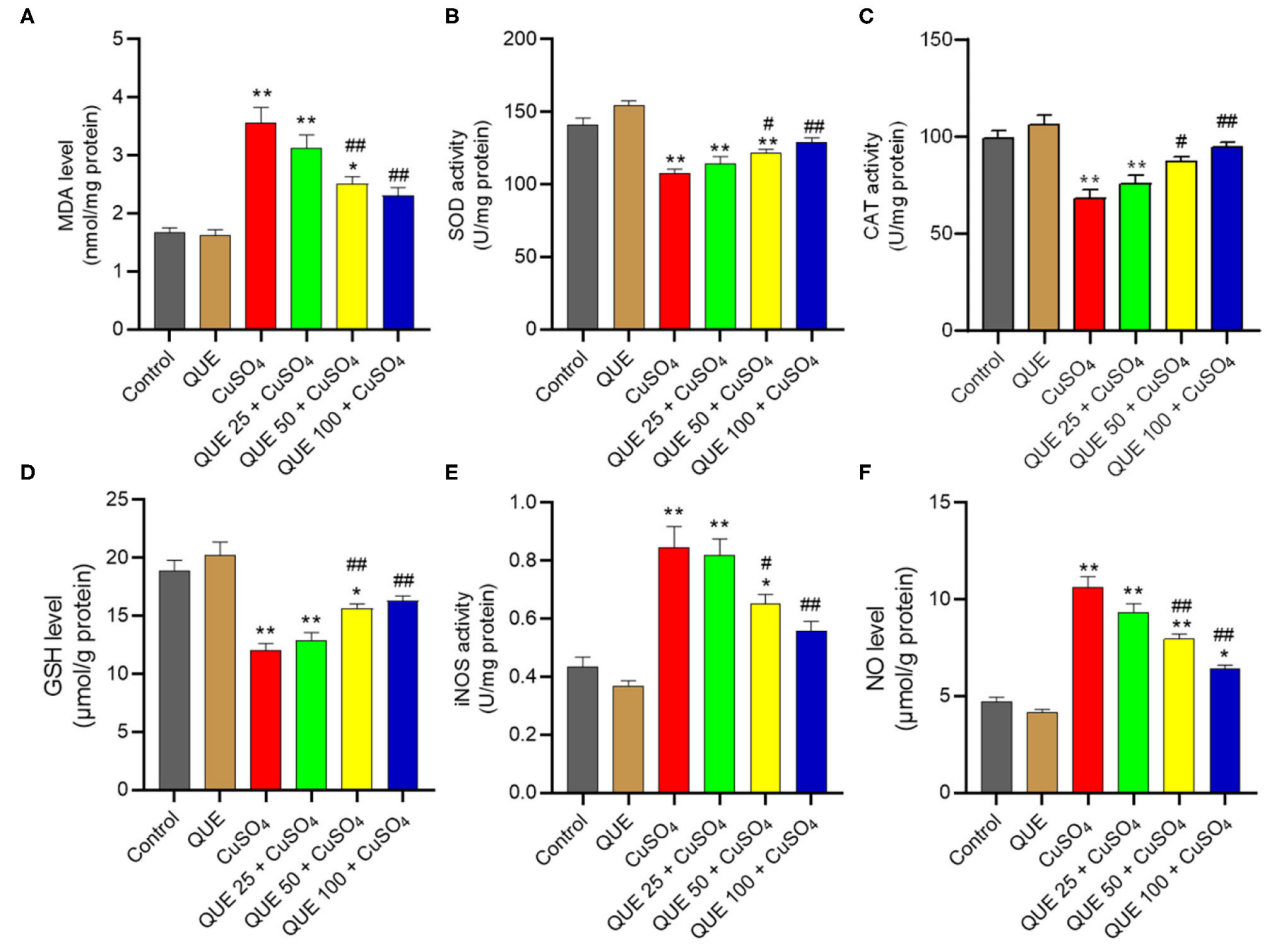
Quercetin supplementation attenuates CuSO_4_-induced oxidative stress in the mouse's kidney tissue. **(A)** malondialdehyde (MDA) levels; **(B)** the activity of superoxide dismutase (SOD); **(C)** the activity of catalase (CAT); **(D)** Glutathione (GSH) levels; **(E)** the activities of inducible nitric oxide synthase (iNOS); **(F)** nitric oxide (NO) levels. Data are reported as mean ± S.E.M (*n* = 10). *P < 0.05, or **P < 0.01, compared to the untreated control group, respectively; ^#^*P* < 0.05, or ^##^*P* < 0.01, compared to the CuSO_4_ alone group, respectively. QUE, quercetin.

CuSO_4_ exposure markedly induced oxidative stress in the kidney tissues of mice (Figure 4). CuSO_4_ exposure significantly increased in the levels of MDA, iNOS and NO to 3.6 nmol/mg protein, 0.84 U/mg protein and 10.6 μmol/g protein (all *P* < 0.01), respectively. It also significantly decreased the activities of SOD and CAT and the level of GSH to 107.7 U/mg protein, 68.1 U/mg protein, and 12.1 μmol/g protein (all *P* < 0.01), respectively. Quercetin supplementation at the doses of 50 and 100 mg/kg/day significantly downregulated the levels of MDA (to 2.5 and 2.3 nmol/mg protein, respectively; Figure 4A), the activities of iNOS (to 0.65 and 0.56 U/mg protein, respectively; Figure 4E) and the levels NO (to 7.9 and 6.4 μmol/g protein, respectively; Figure 4F). Compared to the CuSO_4_ alone group, quercetin supplementation at the doses of 50 and 100 mg/kg/day also significantly upregulated the activities of SOD (to 121.5 and 128.9 U/mg protein, respectively; Figure 4B), CAT (to 87.3 and 94.4 U/mg protein, respectively; Figure 4C), and GSH levels (to 15.6 and 16.3 μmol/g protein, respectively; Figure 4D) (all *P* < 0.05 or 0.01). Quercetin supplementation at 25 mg/kg/day did not significantly attenuate CuSO_4_-induced oxidative stress. In the quercetin alone group, the levels of MDA, NO, GSH and the activities of SOD, CAT, and iNOS showed no significant alterations, compared to that in the kidneys of untreated mice (Figure 4).

### Quercetin Supplementation Attenuated CuSO_4_-induced Expressions in the Levels of IL-1β, TNF-α, and IL-6 Proteins

**Figure 5:**
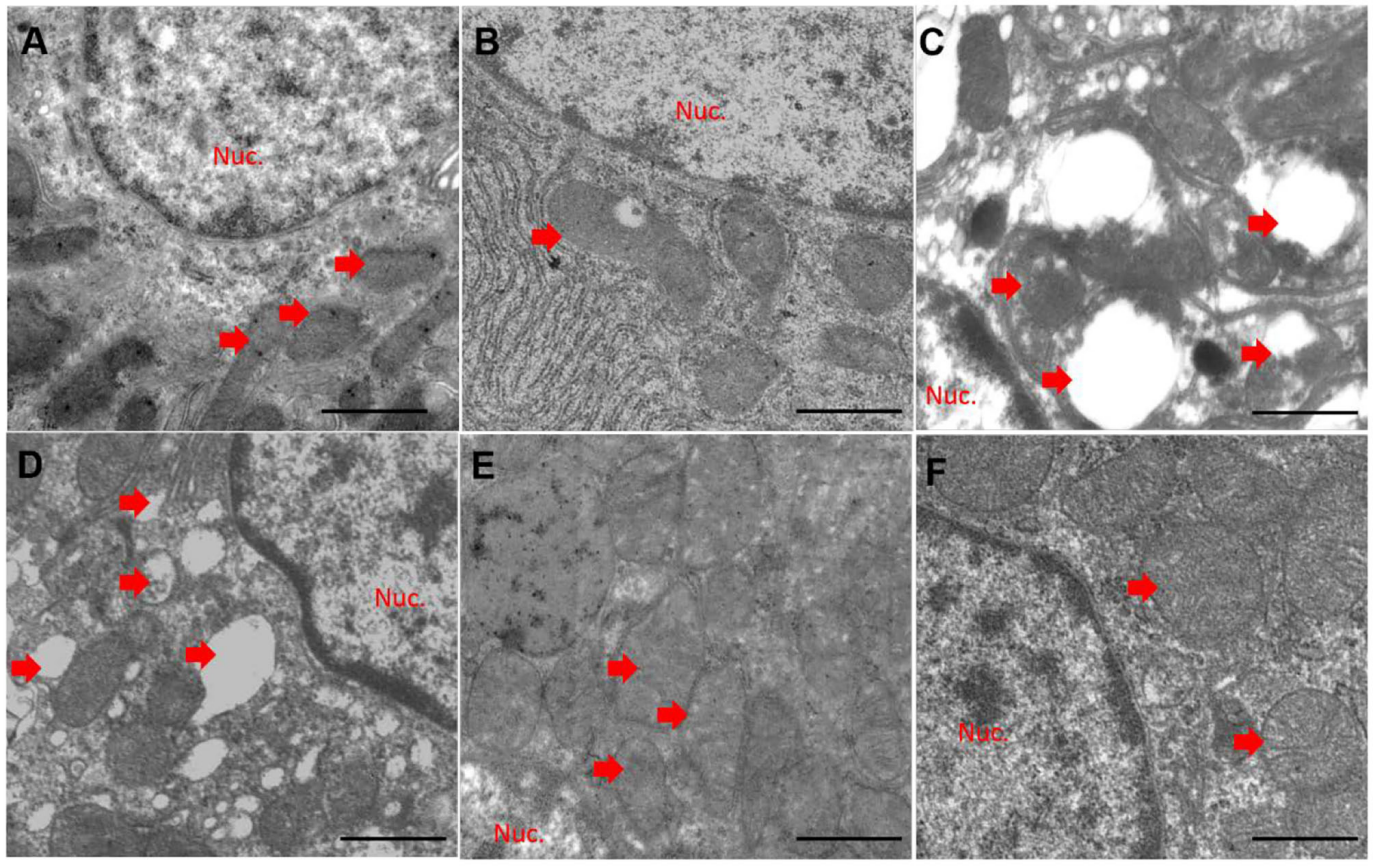
Quercetin supplementation attenuates CuSO_4_-induced mitochondrial damage in the kidney tissues of mice (*n* = 3). The representative mitochondrial ultrastructure in the control **(A)** CuSO_4_ alone **(B)** quercetin (QUE100) alone **(C)** QUE25 + CuSO4 **(D)** QUE50 + CuSO_4_ **(E)** and QUE100 + CuSO_4_ **(F)** groups. Red arrows indicated mitochondria. Nuc, nuclear. Bar = 1μm.

Compared to the control group (Figure 5A), quercetin treatment alone did not significantly alter mitochondrial morphology (Figure 5B). CuSO_4_ exposure led to marked pathological changes in mitochondrial morphology, characterized by the appearance of swollen and ruptured mitochondria, and the disappearance of cristae (Figure 5C). These pathological changes were significantly diminished in the QUE50 + CuSO_4_ and QUE100 + CuSO_4_ groups, but not in the QUE25 + CuSO_4_ group (Figures 5E,F).

### Quercetin Supplementation Attenuated CuSO_4_-induced the Activation of Mitochondrial Apoptosis Pathway in the Kidney Tissues of Mice

**Figure 6:**
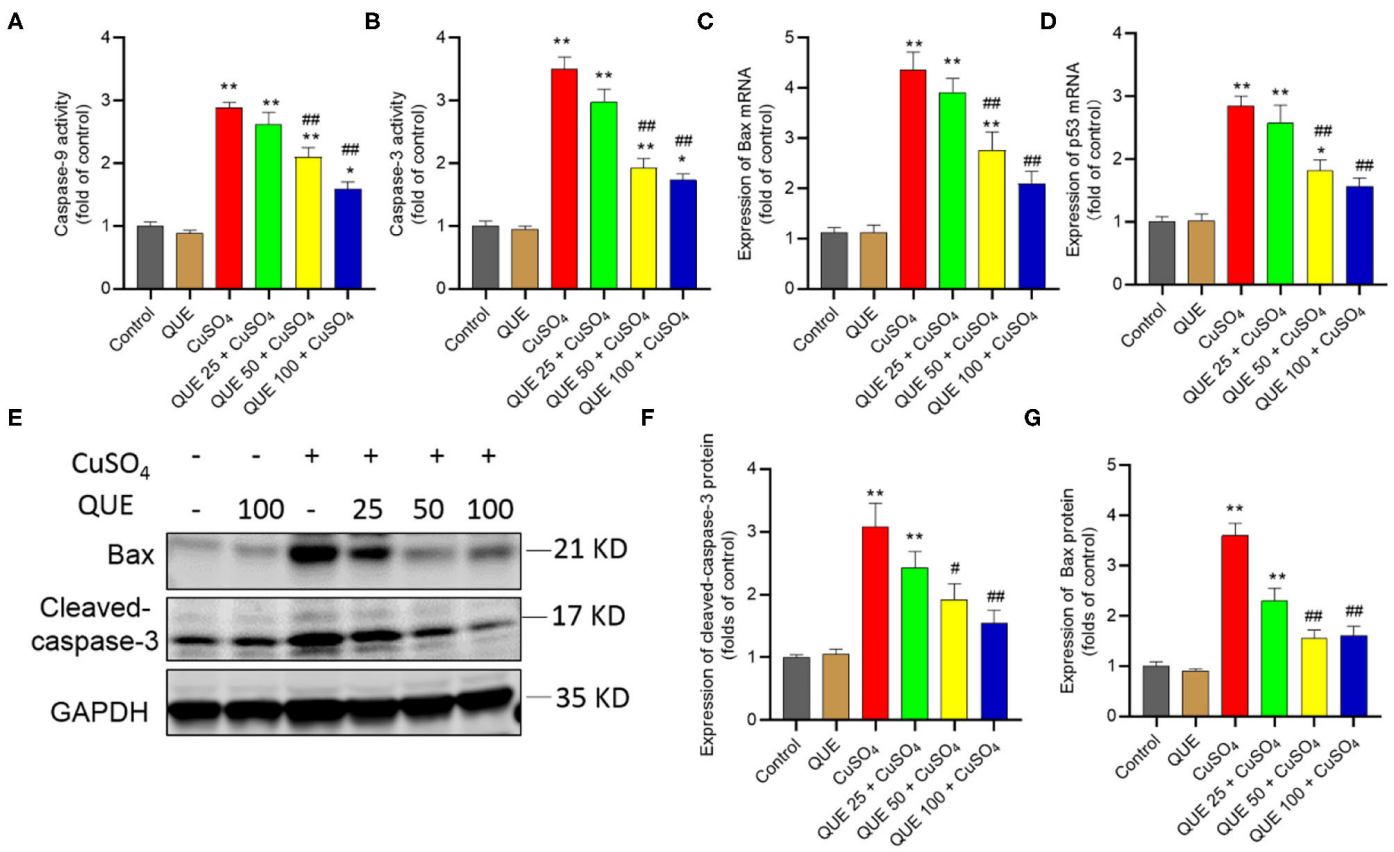
Quercetin supplementation attenuates CuSO_4_-induced the activities of caspases-9,−3, the expression of Bax and p53 mRNAs, and the expression of cleaved caspase-3 and Bax proteins in the mouse's kidney tissue. **(A,B)** the activities of caspases-9 and−3, respectively; **(C,D)** the expression of Bax and p53 mRNAs, respectively; **(E–G)** Representative expression of cleaved caspase-3 and Bax proteins by western blot **(E)** and quantification of cleaved-capase-3 **(F)** and Bax **(G)**. Data are reported as mean ± S.E.M [*n* = 8 in **(A–D)** and *n* = 6 in **(F–G)**]. *P < 0.05, or **P < 0.01, compared to the untreated control group, respectively; ^#^*P* < 0.05, or ^##^*P* < 0.01, the CuSO_4_ alone group, respectively. QUE, quercetin.

When seen against the vehicle control group, CuSO_4_ exposure significantly increased the activity of caspases-9 and−3 (both *P* < 0.01). Compared to the CuSO_4_ alone group, the kidney tissues of mice in the QUE50 + CuSO_4_ and QUE100 + CuSO_4_ groups show the activities of caspase-9 fall from 2.9-fold to 2.1-fold and 1.6-fold, respectively and the activities of caspase-3 fall from 3.5-fold to 1.9-fold and 1.7-fold (all *P* < 0.05 or 0.01), respectively (Figures 6A,B). Quercetin supplementation also decreased CuSO_4_ exposure-induced increases in the expression of Bax and p53 transcripts. In the QUE50 + CuSO_4_ and QUE100 + CuSO_4_ groups, the expression of Bax mRNA decreased from 4.4-fold to 2.8-fold and 2.1-fold, respectively (Figure 6C); the p53 mRNA decreased to from 2.8-fold to 1.8-fold and 1.5-fold, respectively (Figure 6D) (all *P* < 0.05 or 0.01). Furthermore, compared to CuSO_4_ alone group, marked attenuation in the levels of Bax and cleaved caspase-3 were detected in the QUE50 + CuSO_4_ and QUE100 + CuSO_4_ groups (all *P* < 0.05 or 0.01) (Figures 6E–G). These apoptotic markers showed no significant effect in the QUE25 + CuSO_4_ group. Compared to the control, quercetin treatment at 100 mg/kg per day did not significantly affect the activities of caspases-9 and−3, the expression of p53 and Bax mRNAs, and Bax and cleaved caspase-3 proteins (Figures 6A–G).

### Quercetin Supplementation Attenuated CuSO_4_-induced Upregulation in the Levels of IL-1β, TNF-α, and IL-6 Proteins

**Figure 7:**
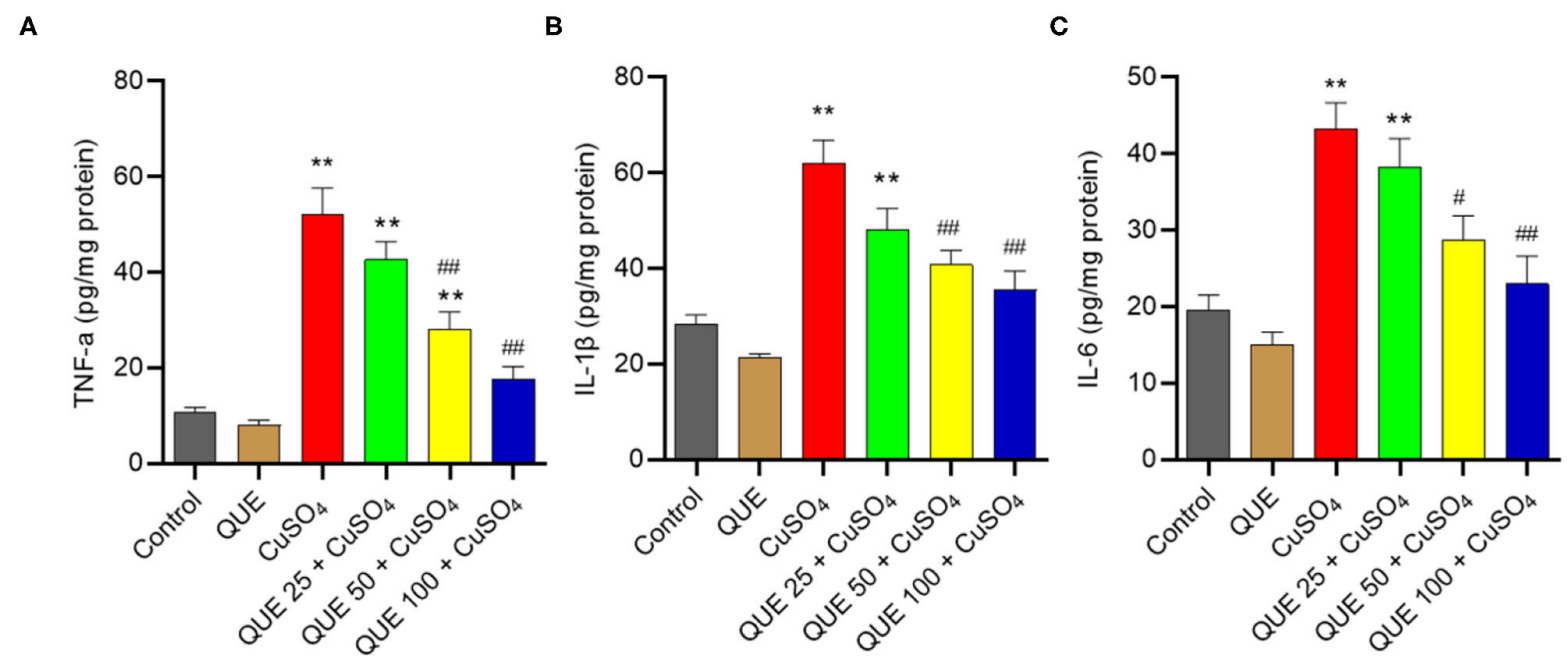
Quercetin supplementation attenuates CuSO_4_-induced the expression of TNF-α, IL-1β, and IL-6 proteins in the kidney tissues of mice. The levels of TNF-α **(A)** IL-1β **(B)** and IL-6 **(C)** proteins in the kidney tissues of mice were measured by using the ELISA method. Data are reported as mean ± S.E.M (*n* = 7). **P < 0.01, compared to the control group, respectively; ^#^*P* < 0.05, or ^##^*P* < 0.01, the CuSO_4_ alone group, respectively. QUE, quercetin.

As shown in Figure 7, CuSO_4_ exposure significantly induced an inflammatory response in the renal tissue of mice, quantified by an upregulation in the levels of TNF-α, IL-6, and IL-1β compared in the control group. Quercetin supplementation, especially at 50 and 100 mg/kg per day (i.e., in the QUE50 + CuSO_4_ and QUE100 + CuSO_4_ groups), markedly attenuated all of these CuSO_4_ exposure-induced expression of inflammatory biomarkers (*P* < 0.05 or 0.01) (Figure 7). A slight decrease in the levels of TNF-α, IL-6, and IL-1β were detected in the QUE25 + CuSO_4_ group, compared to CuSO_4_ alone group, but it was not significant. The levels of TNF-α, IL-1β, and IL-6 did not appreciably change in the quercetin alone group, compared to that in the control group (Figure 7).

### Quercetin Supplementation Regulated the Gene Expressions in Nrf2 and NF-κB Pathways

**Figure 8:**
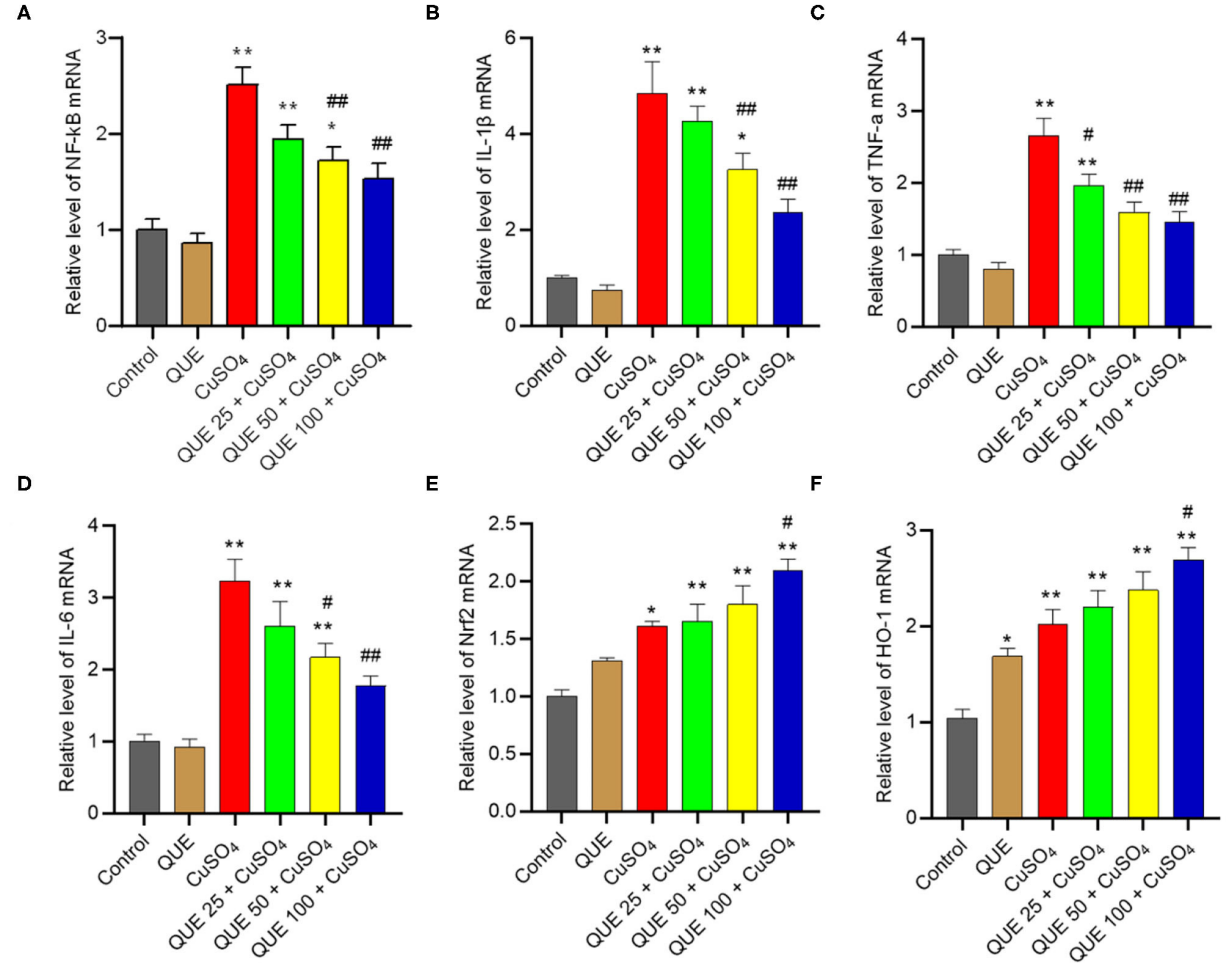
Effect of quercetin supplementation on the expression of NF-κB **(A)**, IL-1β **(B)**, TNF-α **(C)**, IL-6 **(D)**, Nrf2 **(E)**, HO-1 **(F)** mRNAs in the kidney tissues of mice. Data are reported as mean ± S.E.M (*n* = 7). *P < 0.05, or **P < 0.01, compared to the control group, respectively; ^#^*P* < 0.05, or ^##^*P* < 0.01, compared to the CuSO_4_ alone group, respectively. QUE, quercetin.

CuSO_4_ exposure significantly up-regulated the expression of Nrf2, HO-1, NF-κB, IL-1β, TNF-α, and IL-6 mRNAs, compared to that in the kidneys of untreated control group. Quercetin supplementation, especially at 100 mg/kg/day (i.e., QUE100 + CuSO_4_ group), significantly reduced the expression of NF-κB, IL-1β, TNF-α, and IL-6 mRNAs (Figures 8A–D), but increased the expression of Nrf2 and HO-1 mRNAs (all *P* < 0.05 or 0.01) (Figures 8E,F). In the QUE25 + CuSO_4_ group, the mRNA levels of TNF-α, decreased significantly, but not Nrf2, HO-1, NF-κB, IL-1β, or IL-6, were decreased, compared to those in the CuSO_4_ alone group. Quercetin treatment at 100 mg/kg/day alone mildly increased the expression of Nrf2 and HO-1 mRNAs and did not significantly changed the expression of NF-κB, IL-1β, TNF-α, and IL-6 mRNAs, compared to that in the untreated mice.

## Discussion

Copper (Cu) can enter into cells though the copper transporter 1 (27, 28). Excessive Cu accumulation in multiple organs (e.g., brain, kidney, heart, liver, and reproductive organs) due to both acute and chronic uptake causes potential toxic effects, including nephrotoxicity, hepatotoxicity, neurotoxicity and reproductive toxicity (4, 10, 11, 29–31). In line with the previous studies (4), our current study showed that CuSO_4_ exposure at the dose of 200 mg/kg/day for 28 days caused marked nephrotoxicity in mice, observed through the upregulation of serum BUN and CRE levels, as well as histopathological damage (Figures 2, 3). Quercetin is a polyphenolic flavonoid compound found in various vegetables and fruits (32) and it has many documented pharmacologically relevant activities (19, 33, 34). In the present study, our data showed that co-administration of quercetin (at the doses ranging from 25–100 mg/kg/day) significantly attenuated CuSO_4_ exposure-induced increases of BUN and CRE and histopathological damages in a dose-dependent manner, indicating the nephroprotective effect of quercetin (Figures 2, 3).

The previous studies had demonstrated that oxidative stress might play a critical role in CuSO_4_-induced nephrotoxicity *in vitro* in kidney cell lines (i.e., HEK293 cells) and *in vivo* in a mouse/rat nephrotoxicity model (10, 23). In line with these observations, lower levels of GSH and marked decreases of SOD and CAT activities were detected in the kidney tissues of mice exposed with CuSO_4_ (Figure 3). Moreover, CuSO_4_ exposure significantly increased the level of MDA, a lipid peroxidation marker, and nitrative stress-related iNOS activities and NO levels in the kidney tissues of the CuSO_4_-treated mice (Figure 4). The potent anti-oxidant activity of quercetin is related with its –OH groups on the side phenyl ring (35). Pretreatment with quercetin has been shown to increase the levels of endogenous antioxidant enzymes, including Mn-SOD, Cu/Zn SOD, GSH peroxidase and CAT in hippocampal CA1 neurons of gerbils affected by ischemic injury (36). Similarly, a previous study showed that quercetin supplementation at 40 mg/kg/day for 4 weeks markedly reduced cadmium (Cd)-induced nephrotoxicity in rats by up-regulating the anti-oxidant enzymes activities (e.g., SOD and CAT, glutathione peroxidase) and the levels of vitamin C and vitamin E in the kidneys (24). In a rat model, oral supplementation of quercetin at 50 mg/kg/day for 7 days inhibited gentamicin-induced nephrotoxicity via the inhibition of lipid peroxidation and oxidative stress (25). In another study, quercetin supplementation at 50 mg/kg/day in drinking water for 75 days markedly reduced lead-induced renal oxidative damage in rats (37). In our study, supplementation with quercetin, especially at 50 and 100 mg/kg/day, significantly reduced CuSO_4_ exposure-induced adverse oxidative/nitrative changes (Figure 4). Taken together, our results add to the growing body of evidence suggesting that quercetin supplementation could protect against nephrotoxicity in mice by inhibiting oxidative stress and nitrative stress.

Copper ions exist as Cu^2+^ (oxidized) or Cu^1+^ (reduced) forms in biological systems (38). This process is homeostatic under normal physiological conditions. Cu overdose could disturb this balance and induce the production of excessive ROS, which leads to lipid, protein and DNA damage (39). Previous studies demonstrated that CuSO_4_ or copper chloride (CuCl_2_) exposure can induce the production of excessive ROS in HEK293 cells, mouse liver cells or neuronal cells (23, 40, 41). Mitochondria are not only the major producer of cellular ROS but also a target (42). It has been demonstrated that CuSO_4_ exposure can cause mitochondrial dysfunction in neuroblastoma SH-SY5Y cells, and human hepatoma (HepG2) cells (43, 44). In the present study, CuSO_4_ exposure caused visible mitochondrial damage, characterized by swollen and ruptured mitochondria and disappearance of cristae. These pathological changes were reduced by quercetin supplementation in a dose-dependent manner (Figure 5). Caspase-9 and Bax are two important biomarkers of the mitochondrial apoptotic pathway (45). Caspase-3 and p53 activation are key biomarkers of apoptosis (39). p53 has the ability to directly or indirectly activate Bax (46). In the present study, CuSO_4_ exposure significantly upregulated the activities of capases-9 and−3, and the expression of p53, Bax and cleaved-caspase-3 (Figure 6). A previous study also showed that quercetin supplementation significantly reduced the expression of cleaved caspase-3 and apoptosis in the kidney tissues and improved cisplatin nephrotoxicity in mice (47). Recently, Pakrashi et al. (48) also showed the protective effect of quercetin on ROS induced mitochondrial dysfunction in a rotenone-induced apoptotic model. Our study shows that quercetin supplementation decreased the expression of these pro-apoptotic genes and markedly suppressed these adverse indications (Figure 6). The results collectively indicate that CuSO_4_-exposure induced renal toxicity involved the activation of mitochondrial apoptotic pathway and inhibition of this pathway partially contributed to the nephroprotective effect of quercetin.

Nrf2 is a transcription factor that regulates the expression of anti-oxidant enzymes, including CAT, SOD, and HO-1(49). The Nrf2 pathway is important in the process of cytoprotective adaptive responses to xenobiotic exposure (50). Nrf2 is highly active in tissues or cells susceptible to oxidative stress from exposure to drugs or toxins (50–52). Under normal conditions, the Nrf2 activity is suppressed, as it is sequestered in the cytoplasm by binding to Kelch-like ECH-associated protein 1 (Keap1) (50). It has been reported that CuSO_4_ exposure can induce the expression of Nrf2 and downstream gene HO-1 in Hela, HEC-1A, HEK293, and A549 cells (16). In the present study, CuSO_4_ significantly increased the expression of Nrf2 and HO-1 expression in the kidney tissues (Figures 7E,F). Quercetin can directly interact with the binding site of Nrf2 in Keap1 protein and activate the transcriptional activity of Nrf2 (53). Consistently, quercetin supplementation *per se* further upregulated the expressions of Nrf2 and HO-1 and further promoted their expressions in the kidneys exposed with CuSO_4_ at 200 mg/kg per day for 28 days. Taken together, the enhanced activation of Nrf2/HO-1 pathway may contribute to the nephroprotective effect of quercetin.

Inflammation plays a critical role in the progression of CuSO_4_ exposure-induced toxic effects (54–56). In the present study, our results showed that CuSO_4_ exposure significantly upregulated the expression of IL-1β, TNF-α, and IL-6 proteins and mRNAs in the kidney tissues. Quercetin supplementation significantly inhibited the expression of IL-1β, IL-6, and TNF-αproteins and mRNAs. It thus appears that quercetin supplementation could reduce CuSO_4_ exposure-induced inflammatory response in the kidney tissues (Figures 7, 8). NF-κB is a master transcriptional mediator that plays a critical role in the cell responding to a diverse set of inflammatory stimuli (49). NF-κB is known to mediate the expression of more than 500 genes, including IL-6, IL-1β, TNF-α, and iNOS (57). Sanchez-Gonzalez and colleagues demonstrated that quercetin supplementation inhibited cisplatin induced renal damage in rats by inhibiting the expression of NF-κB and iNOS protein (47). A study by Liu et. al. demonstrated that quercetin supplementation protected against lead-induced nephrotoxicity in the rat kidney through the inhibition of inflammatory response by downregulating the mitogen-activated protein kinase (MAPKs) and NF-κB pathways. In the present study, our results also showed that CuSO_4_ exposure significantly upregulated the expression of NF-κB and iNOS activities and NO levels, which were inhibited significantly by quercetin supplementation (Figures 4, 8). Thus, these observations indicate that the inhibitory effect of quercetin on the inflammation caused by CuSO_4_ was attributed partially to the inhibition of NF-κB/iNOS/NO pathway. In addition, accumulation of ROS could activate NF-κB pathway (58). It is not clear from our study whether the production of ROS caused by CuSO_4_ exposure contributed to the activation of NF-κB. That would warrant further investigation.

A growing body of evidence indicates that high levels of free copper has a targeted effect on oxidation of hemoglobin, as well as direct damage to the cell membrane, which may contribute to its nephrotoxicity (59). Several studies have reported that quercetin supplementation could inhibit the oxidation of hemoglobin via the Fenton pathway (60) or alleviate the toxic effects of bisphenol A in human red blood cells. It is not clear that whether quercetin supplementation can affect Cu-caused hemolysis effect (61). This area still needs further investigation.

## Conclusions

**Figure 9:**
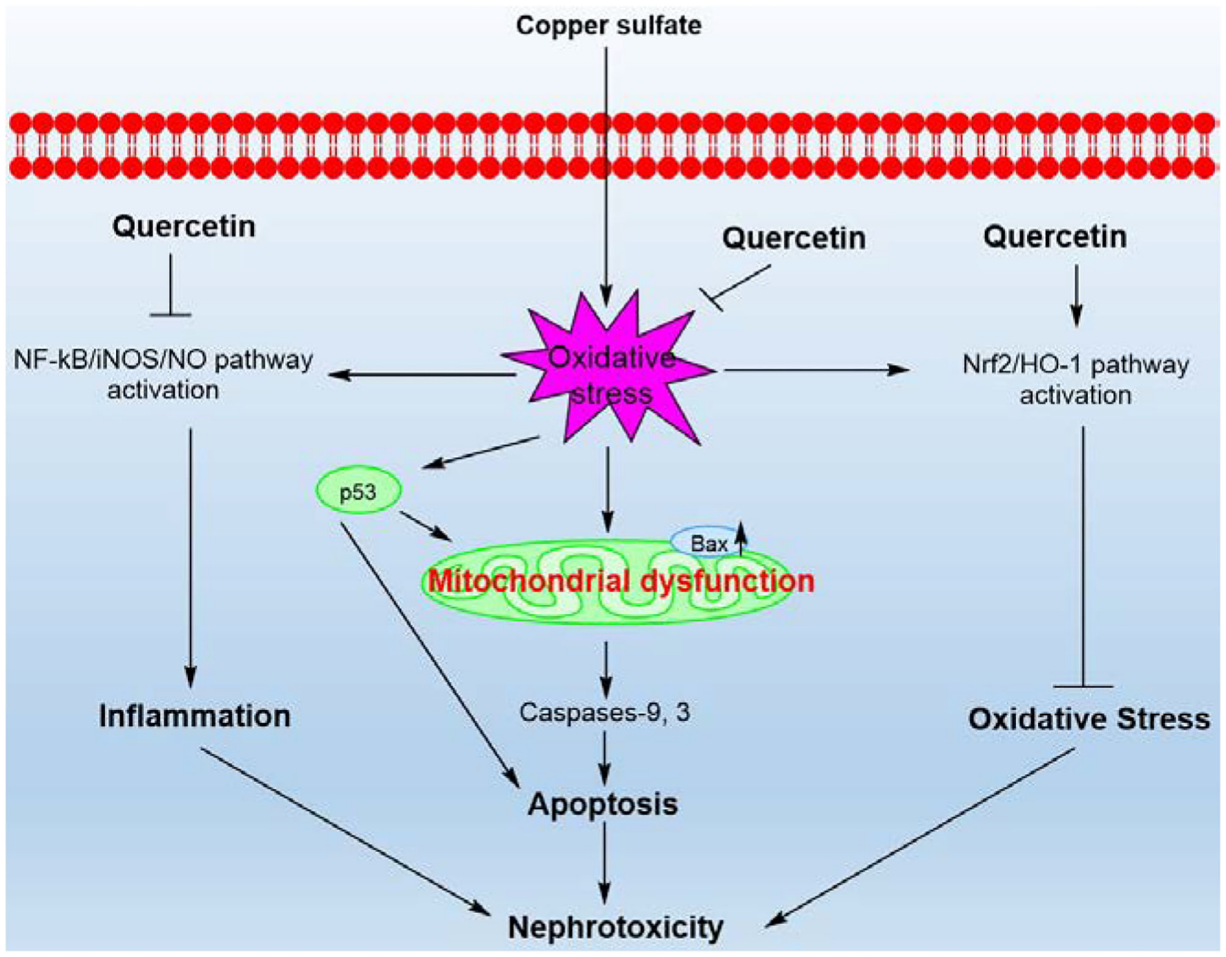
A proposed working model highlighting the protective effects of quercetin on CuSO_4_-exposure induced nephrotoxicity. CuSO_4_ exposure triggers oxidative stress, mitochondrial apoptotic pathway, Nrf2/HO-1 pathway and NF-κB/iNOS/NO pathway in the mouse's kidney tissue. CuSO_4_ exposure also increased the expression of p53, which may contribute to the activation of Bax and exacerbate mitochondrial dysfunction. Quercetin supplementation could inhibit oxidative stress, NF-κB/iNOS/NO and mitochondrial apoptotic pathways and activate the Nrf2/HO-1 pathway, thereby ameliorating CuSO_4_-induced nephrotoxicity in mice.

This study revealed that CuSO_4_ exposure could trigger the activation of p53 pathway, mitochondrial pathway, and oxidative stress and NF-κB mediated-inflammatory response in the kidney tissues, which subsequently lead to significant nephrotoxicity in mice (Figure 9). Quercetin supplementation could effectively attenuate CuSO_4_-exposure induced nephrotoxicity by reducing oxidative stress, apoptosis and inflammatory responses. Its protective effects involve the inhibition of mitochondrial apoptotic and NF-κB pathways and the activation of Nrf2/HO-1 pathway. Our findings highlight that quercetin may be a promising therapeutic agent against Cu exposure- induced nephrotoxicity.

## Data Availability Statement

The raw data supporting the conclusions of this article will be made available by the authors, without undue reservation.

## Ethics Statement

The animal study was reviewed and approved by The Institutional Animal Care and Use Committee at the Ludong University.

## Author Contributions

XP and CD: conceptualization, methodology, and original manuscript draft. CD: software. XP, MZ, and CD: formal analysis. XP and MZ: investigation. XP: data curating, acquisition of funding, and project management. XP, SD, MZ, and CD: draft review. All authors have viewed the manuscript and agree to its publication. All authors contributed to the article and approved the submitted version.

## Conflict of Interest

The authors declare that the research was conducted in the absence of any commercial or financial relationships that could be construed as a potential conflict of interest.
